# 间质性肺病合并肺癌的外科治疗

**DOI:** 10.3779/j.issn.1009-3419.2026.106.14

**Published:** 2026-05-20

**Authors:** Yang LIU, Chuan HUANG

**Affiliations:** 100730 北京，北京医院胸外科，国家老年医学中心，中国医学科学院老年医学研究院; Department of Thoracic Surgery, Beijing Hospital, National Center of Gerontology, Institute of Geriatric Medicine,Chinese Academy of Medical Sciences, Beijing 100730, China

**Keywords:** 间质性肺病, 肺肿瘤, 外科治疗, 围术期管理, 急性加重, Interstitial lung disease, Lung neoplasms, Surgical treatment, Perioperative management, Acute exacerbation

## Abstract

间质性肺病（interstitial lung disease, ILD）以肺间质不同程度的炎症和纤维化为特点，严重威胁人类健康。ILD患者罹患肺癌的风险显著高于普通人群，这使得间质性肺病合并肺癌（ILD combined with lung cancer, ILD-LC）逐渐受到关注。目前，外科治疗是ILD-LC患者的首选治疗方式。然而，如何为此类患者制定个体化的手术策略亟需探讨。此外，ILD-LC患者术后易发生ILD急性加重（acute exacerbation of ILD, AE-ILD），且不易早期识别，严重影响预后。基于上述背景，本文系统综述了ILD-LC的鉴别诊断、手术策略、围术期管理等内容，尤其介绍术后AE-ILD的识别及处理策略，以期为ILD-LC的外科治疗提供参考，并对未来的研究方向进行展望。

## Abstract (en)

Interstitial lung disease (ILD) is characterized by varying degrees of inflammation and fibrosis of the pulmonary interstitium, posing a significant threat to human health. Patients with ILD have a significantly higher risk of developing lung cancer compared to the general population, which has led to growing clinical attention to the condition of ILD combined with lung cancer (ILD-LC). Nowadays, surgical intervention remains the primary treatment option for patients with ILD-LC. However, how to formulate individualized surgical strategies for this patient population represents a critical challenge in clinical practice. Additionally, patients with ILD-LC are prone to postoperative acute exacerbation of ILD (AE-ILD), which is often challenging to detect at an early stage and severely compromises prognosis. Against this background, this article systematically reviews key aspects of ILD-LC, including differential diagnosis, surgical strategies, and perioperative management, with particular emphasis on the recognition and management of postoperative AE-ILD. It aims to provide a reference for the surgical treatment of ILD-LC and to outline directions for future research.

间质性肺病（interstitial lung disease, ILD）是一类异质性的弥漫性肺间质疾病的总称，涉及肺间质不同程度的炎症和纤维化，引起限制性通气功能障碍和弥散功能障碍，严重威胁人类健康^[1]^。对ILD进行分类，需要评估环境、职业及其他暴露史（如胸部放疗史、用药史），并排查与ILD可能相关的系统性疾病（如系统性红斑狼疮、类风湿性关节炎等）。

近年来，随着胸部计算机断层扫描（computed tomography, CT）在肺部体检中的广泛应用，在肺间质性病变基础上检出肺结节的临床场景逐渐增加，ILD合并肺癌（ILD combined with lung cancer, ILD-LC）也愈加受到关注^[2]^。ILD患者罹患肺癌的风险显著增加，ILD-LC发生率可达10%-20%^[3,4]^。因此，对ILD-LC进行早期鉴别意义重大。多数ILD病情进展缓慢，但在肺切除、肺活检、支气管镜检查等诱因下可出现ILD急性加重（acute exacerbation of ILD, AE-ILD）。AE-ILD是ILD-LC患者围手术期较常见和严重的并发症之一，发生率可达12%-27%，且预后极差，死亡率达33%-100%^[5,6]^。如何识别AE-ILD的发生风险并降低术后AE-ILD的发生率亦是胸外科医师亟需探讨的重要议题。因此，对于ILD-LC患者来说，早期、及时的鉴别诊断，个体化的手术策略和精细化的围手术期管理尤为重要，是实现良好的肿瘤学远期预后的基础。本文旨在通过系统梳理相关文献，阐述ILD-LC患者的鉴别诊断、手术策略、围手术期管理等内容，尤其介绍术后AE-ILD的识别及处理策略，以期为ILD-LC的外科治疗提供有益借鉴。

## 1 ILD-LC的流行病学及发病机制

ILD是一类以肺间质慢性炎症和纤维化为特征的疾病总称^[1]^。按照病因将ILD进行分类，其中约60%为病因不明的特发性ILD，另有40%为继发性ILD。特发性ILD包括特发性肺纤维化（idiopathic pulmonary fibrosis, IPF）、特发性非特异性间质性肺炎（idiopathic nonspecific interstitial pneumonia, iNSIP）、隐源性机化性肺炎（cryptogenic organizing pneumonia, COP）、急性间质性肺炎（acute interstitial pneumonia, AIP）、呼吸性细支气管炎伴间质性肺疾病（respiratory bronchiolitis-associated ILD, RB-ILD）、脱屑性间质性肺炎（desquamative interstitial pneumonia, DIP）等^[1]^。在继发性ILD中结缔组织疾病相关的ILD（connective tissue diseases related ILD, CTD-ILD）近年来广受关注。此外，系统性红斑狼疮、类风湿性关节炎、干燥综合征等均可累及肺部，引发ILD^[7]^。

流行病学数据^[8]^显示，ILD在总人群中的发病率为6.3/10万-76/10万，其分布存在年龄、性别、种族及地域差异。IPF好发于老年男性，在欧洲和北美洲的发病率为0.9/10万-9.3/10万，在亚洲和南美洲为3.5/10万-13.0/10万^[9,10]^。与IPF不同，50%以上的iNSIP和CTD-ILD患者为中年或老年女性^[11-13]^。其他类型ILD的流行病学数据相对有限，现有报道^[9,13]^提示其发病率普遍低于IPF。

肺癌是目前我国乃至世界范围内发病率居于首位的恶性肿瘤，也是造成全球癌症相关死亡的主要原因^[14,15]^。值得注意的是，ILD患者罹患肺癌的风险显著增高，约为普通人群的7-14倍^[16]^。统计数据^[3,4]^显示，各类型ILD罹患肺癌的总发病率为10%-20%，风险比（odds ratio, OR）为3.52，其中IPF患者中LC的发病率达13%-20%，OR达4.99。IPF患者的肺癌风险随着IPF的病程逐年增加，病程为1年的患者肺癌发病率为1.1%-5.5%，5年时升至12.2%-15.9%，10年时则高达26.6%-54.7%^[17]^。

肺腺癌（lung adenocarcinoma, ADC）是普通人群中最常见的肺癌病理类型，而在ILD患者群体中，肺鳞状细胞癌（lung squamous cell carcinoma, LSCC）最为常见，占所有ILD-LC患者的33.3%-39%^[17-19]^。此外，肺癌在普通人群中以上叶多见，而ILD-LC的肿瘤病灶多位于外周肺，常见于肺下叶（48.6%-58.7%）^[20]^。

ILD与肺癌关联的发病机制尚未完全阐明。有研究^[21-23]^认为ILD和肺癌之间可能存在共同的病理生理过程，如上皮细胞的异常变化、可溶性介质释放、基因变异以及异常信号通路的激活等，高龄、男性、表皮生长因子受体（epidermal growth factor receptor, EGFR）野生型突变均是ILD患者罹患肺癌的危险因素。另有研究^[24,25]^证实，吸烟是ILD和肺癌的共同危险因素，可显著增加二者的发生风险。

## 2 ILD-LC的鉴别诊断

高分辨CT（high-resolution CT, HRCT）可在ILD患者随访期间实现肺癌的早期检测^[26]^。然而，IPF-LC患者的肿瘤病灶多位于或临近纤维化区域，难以从CT图像上进行有效区分^[27]^。此外，无论是IPF或肺癌患者，其纵隔淋巴结都有肿大的可能。因此，HRCT中纵隔淋巴结肿大的表现在IPF和肺癌之间没有显著特异性^[28]^。基于上述特点，ILD患者在随访过程中若胸部CT有任何新发现的肺占位表现，均应高度警惕ILD-LC，并做全面的鉴别诊断^[29]^。

正电子发射断层显像/CT（positron emission tomography/CT, PET/CT）在鉴别IPF和肺癌方面具有良好的效能。一项回顾性研究^[30]^纳入了55例IPF患者，发现PET/CT在识别其所患肺结节良恶性的特异性和敏感性分别为86%和98%。此外，在IPF-LC患者的纵隔淋巴结分期方面，PET/CT也显示出优于CT的诊断价值，其特异性（91% vs 47%, P=0.0002）和敏感性（83% vs 50%, P=0.001）均明显更高^[31]^。

若PET/CT提示肺结节可疑恶性，则需进行活检进一步明确诊断，可选方式包括CT引导下经胸穿刺活检（CT-guided transthoracic needle biopsy, CT-TNB）或支气管镜超声引导下活检^[32]^。由于IPF患者的肺结节病灶多位于外周，所以相较于支气管镜超声引导下活检，CT-TNB更适用于鉴别此类结节的良恶性。一项回顾性研究^[33]^报道CT-TNB用于鉴别IPF合并肺结节的敏感性和特异性分别为90%和84%，但穿刺相关并发症率较高[气胸8.7%、特发性肺纤维化急性加重（acute exacerbations of idiopathic pulmonary fibrosis, AE-IPF）2%]。因此，行CT-TNB前需对IPF的程度充分评估，最大限度避免发生AE-IPF等严重并发症。若无法对肺结节进行活检，则应进行多学科评估，选取个体化的诊疗方案^[34]^。然而，目前尚缺乏IPF合并肺结节患者的真实世界研究数据以支持诊断决策。

血清肿瘤标志物或可在鉴别ILD-LC中起到一定作用^[35]^。ILD-LC患者的癌胚抗原（carcinoembryonic antigen, CEA）、糖类抗原（carbohydrate antigen, CA）199、CA125、CA242、细胞角质蛋白19（cytokeratin 19 fragment, CYFRA21-1）、神经元特异性烯醇化酶（neuron-specific enolase, NSE）和鳞状细胞癌抗原（squamous cell carcinoma antigen, SCC）等肿瘤标志物的水平均明显高于单纯ILD及单纯肺癌患者。因此，当ILD患者发现上述肿瘤标志物水平升高时，应高度警惕ILD-LC的可能，并及时随访其变化。

## 3 ILD-LC的外科治疗策略

目前手术是肺癌的主要治疗方式，具有潜在治愈的可能^[36]^。2015年一项大型回顾性研究^[37]^对1763例接受手术治疗的ILD-LC患者进行长期随访，病理IA、IB、IIA、IIB、IIIA、IIIB和IV期患者的5年总体生存（overall survival, OS）率分别为59%、42%、43%、29%、25%、17%和16%。2021年国内另一项真实世界研究^[38]^亦得出了相似的结论，手术目前仍是ILD合并早期肺癌的治疗首选。

ILD的共存是肺癌患者的一个独立且不利的预后因素^[39]^。与接受手术治疗的单纯肺癌患者相比，ILD-LC患者术后5年OS率明显更差（66% vs 78.8%, P=0.007），这或与其术后易出现AE-ILD等肺部并发症和肿瘤高复发率有关^[37,40]^。一项荟萃分析^[41]^显示，相较于单纯肺癌患者，ILD-LC患者OS显著缩短（HR=2.01, 95%CI: 1.71-2.36）。此外，不同类型的ILD-LC患者术后OS差异较大，其中IPF-LC的远期预后显著差于其他非IPF类型的ILD-LC患者^[42]^。另有研究^[39]^发现，ILD显著增加了肺癌患者胸膜侵犯的发生率（49.0% vs 24.5%, P<0.0001），这可能与其肿瘤病灶更邻近胸膜有密切关系。

目前研究^[42]^认为，对于接受手术治疗的ILD-LC患者，显著影响OS的因素除年龄、肿瘤分期外，尚有一氧化碳弥散量（diffusing capacity of the lung for carbon monoxide, DLCO）、肺活量、术后AE-ILD等。对于预测肺活量百分比≤80%的IA期肺癌患者，5年OS率仅为20%；而对于预测肺活量百分比>80%的患者，5年OS率为64.3%（P<0.0001）^[37]^。此外，术前需氧气吸入被认为与ILD-LC患者电视辅助胸腔镜手术（video-assisted thoracoscopic surgery, VATS）术后死亡率增加有关（OR=5.21, 95%CI: 1.19-22.95, P=0.03）^[43]^。除与ILD相关的风险因素外，急诊手术（OR=24.00, 95%CI: 2.63-218.95, P=0.005）、麻醉时间长（OR=1.60, 95%CI: 1.14-2.22, P=0.006）等可显著增加肺炎等肺部并发症风险^[44]^。

考虑到ILD患者手术的高风险，应仔细规划肺切除术的时机和方法。Sato等^[37]^的研究认为，对于ILD合并IA期肺癌的患者，楔形切除术的预后明显差于肺段及肺叶切除术，5年OS率分别为29.2%、60.0%和68.6%。另一项研究^[45]^表明，ILD合并I期肺癌行肺叶切除术的5年OS率明显优于亚肺叶切除术（73.2% vs 46.3%, P=0.036）。因此，上述研究认为对于可耐受肺叶切除的患者，无需刻意限制肺切除范围。对此，一项荟萃分析^[46]^纳入了10项比较肺叶切除术和亚肺叶切除术在ILD-LC患者预后差异的研究，发现两组之间的OS率没有显著差异（HR=0.978, 95%CI: 0.521-1.833, P>0.05），但是亚肺叶切除可显著降低术后AE-ILD风险（OR=0.521, 95%CI: 0.339-0.803, P=0.0031）。目前，一项III期临床研究（JCOG1708）^[47]^正在进行，以评估IPF-LC患者行亚肺叶切除与肺叶切除的远期预后差异。

因此，在为ILD-LC患者制定手术方案时，应兼顾肿瘤学根治原则与全身病情，谨慎选择肺切除范围。结合文献和本中心经验，推荐如下：对于低风险、ILD病情轻且心肺功能良好的患者，应首选根治性肺叶切除术及系统性淋巴结清扫；但对于高风险、ILD病情严重或心肺功能显著降低的患者，选择亚肺叶切除术（肺段切除术优于楔形切除术）更为恰当，可避免因创伤过大导致围术期风险显著升高。

## 4 ILD-LC的围术期管理

术前肺功能检查在评估ILD的严重程度及预后中起到重要作用。对于接受手术治疗的ILD-LC患者，术前用力肺活量（forced vital capacity, FVC）和DLCO的下降显著增加术后急性呼吸窘迫综合征（acute respiratory distress syndrome, ARDS）发生率（13% vs 1.8%, P<0.01）和术后死亡率（8% vs 1.4%, P<0.01）^[48]^。Ley等^[49]^基于年龄、性别、FVC和DLCO构建了GAP评分量表，经证实可预测ILD患者的死亡率和严重程度。Ryerson等^[50]^在此基础上将ILD的各亚型纳入评分，开发了改良的ILD-GAP评分量表，以预测不同ILD亚型患者的预后。此量表的ILD亚型包含以下4类：IPF、慢性过敏性肺炎（chronic hypersensitivity pneumonitis, cHP）、CTD-ILD和iNSIP。然而，在接受手术治疗的ILD-LC患者群体中，GAP评分量表是否具有较好的预测效能尚需进一步探讨。

相较于非ILD的肺癌患者，ILD-LC患者术后发生肺部并发症的风险显著升高，如AE-ILD、肺不张、气胸和肺栓塞等，其中以AE-ILD最为凶险^[51]^。AE-ILD最常见于IPF，也可见于CTD-ILD、iNSIP等其他类型ILD。AE-ILD可发生于IPF的任何阶段，即使在早期或肺功能良好的患者中也可发生^[52]^，肺切除、肺活检、支气管镜检查、支气管肺泡灌洗、放射治疗及部分药物等均可诱发^[53]^。IPF-LC患者术后AE-IPF的发生率可达12%-27%，多见于术后第2-10天，不仅累及术侧肺，更有超过60%患者累及双侧肺，预后极差，死亡率高达33%-100%^[54]^。

AE-IPF典型症状为快速进展的呼吸困难，肺部可闻及吸气末爆裂音（Velcro啰音），胸部CT表现为在原有IPF影像改变基础上出现新的、双肺分布的磨玻璃影或实变影。自1993年日本学者Kondoh提出AE-IPF的概念，其定义和诊断标准历经多次修订^[55]^。2016年AE-IPF国际工作小组修订其诊断标准如下：（1）有IPF病史或目前临床、影像和/或病理学符合IPF诊断标准；（2）近1个月内出现无法用其他原因解释的呼吸困难加重或肺功能恶化；（3）IPF典型CT表现基础上出现新的双侧磨玻璃影和/或实变影；（4）排除左心衰、肺栓塞和其他原因引起的急性肺损伤；（5）无法满足上述诊断标准时，定义为可疑AE-IPF^[53]^。

既往研究^[37,54]^表明，男性、AE-IPF病史、肺切除范围大、胸部CT有肺纤维化的典型表现、血清涎液化糖链抗原-6（Krebs Von den Lungen-6, KL-6）>1000 U/mL、第一秒用力呼气容积（forced expiratory volume in one second, FEV_1_）≤80%、肺弥散功能降低等因素均增加肺切除术后AE-IPF风险，且行肺叶切除、全肺切除术后AE-IPF发生率明显高于楔形切除术。此外，手术时长超过4 h、术中补液量过多及围术期的误吸或可成为术后AE-ILD的发生诱因^[56,57]^。

需要注意的是，肺切除术后AE-ILD的诊治难点之一在于不易早期发现。由于术后肺容积减少、气道分泌物阻塞、肺不张、手术部位疼痛、循环容量过负荷等诸多原因，肺切除术后患者多有咳嗽、胸闷、气短、呼吸困难等症状，其临床表现与AE-ILD很类似，病程早期难以鉴别。而AE-ILD病情进展迅速，若不能及时诊断和干预，患者往往快速进展至呼吸衰竭、需机械通气，而此时难以及时行胸部CT检查以协助诊断，易延误最佳治疗时机。高龄患者行机械通气后又极易继发肺部感染，导致难以鉴别，更增加了大剂量激素治疗的风险。

基于其难以早期识别的特点，多项研究致力于探索可预测AE-IPF发生风险及预后的标志物。血清中晚期糖基化终末产物受体（receptor for advanced glycation end product, RAGE）的可溶性异构体（sRAGE）、5羟-二十碳四烯酸（5-hydroxyeicosatetraenoic acid, 5-HETE）、Toll样受体4的可溶性异构体（soluble Toll-like receptor 4, sTLR4）或可成为预测AE-IPF的新型标志物^[58-60]^。此外，术前胸部CT上出现累及范围超过肺野5%的磨玻璃影、任何形式的实变影、肺动脉干内径>28 mm可成为预测AE-IPF的指标^[61]^。另有研究^[62]^认为，ILD-LC患者若术前PET/CT提示肿瘤病灶SUVmax>2.55，则术后发生AE-ILD的风险更高。

一旦确诊AE-ILD，临床上常用糖皮质激素治疗，如激素冲击或高剂量激素治疗^[63]^。然而，关于激素的用量、用法和疗程，国内外至今尚未达成共识^[64]^。一项最新的荟萃分析^[65]^认为，激素的使用应遵从个体化原则，针对不同的AE-ILD亚型，选择合适的用法及剂量。对于IPF患者，高剂量激素治疗（泼尼松≥1 mg/kg/d）无法带来明确的生存获益。而对于CTD-ILD、iNSIP、HP等其他类型的ILD患者，高剂量激素治疗可显著改善其生存率（HR=0.221, 95%CI: 0.102-0.480, P<0.001）。此外，激素的早期减量（2周内减量>10%）可有效降低此类患者的住院死亡率（HR=0.37, 95%CI: 0.14-0.99）。

传统的观点认为，激素效果不佳时可加用免疫抑制剂如环磷酰胺、他克莫司、环孢素A等。然而，一项多中心的III期临床试验^[66]^结果表明，静脉环磷酰胺冲击疗法联合糖皮质激素会显著增加AE-ILD患者的3个月死亡率。

研究结果表明，围术期应用吡非尼酮对于预防AE-ILD也起到一定的作用。吡非尼酮是吡啶酮类口服合成化合物，通过下调转化生长因子表达、抑制促纤维化因子转录发挥抗纤维化作用^[67]^。手术联用吡非尼酮可显著降低IPF患者围术期急性加重的风险^[68,69]^。一项II期临床研究（WJOG6711L）^[68]^纳入了39例IPF-LC患者，给予口服吡非尼酮600 mg/d，2周后增加剂量至1200 mg/d，按此剂量使用至少2周后进行手术，术后继续按照1200 mg/d的剂量口服吡非尼酮，研究发现94.9%的患者在术后30 d内未出现AE-IPF。另有一项多中心随机对照III期临床研究（NEJ034）^[69]^正在进行，干预组给予口服吡非尼酮后手术，给予剂量及用法与WJOG6711L研究相同，对照组在围术期则不接受任何抗纤维化药物治疗。本研究的主要终点为术后30天内未发生AE-IPF的患者比例，期待其结果进一步证实吡非尼酮降低IPF-LC患者围术期急性加重风险的临床价值。尼达尼布是一种小分子酪氨酸激酶抑制剂，在早期曾作为非小细胞肺癌（non-small cell lung cancer, NSCLC）的一线用药。后续的研究^[70]^发现尼达尼布具有显著的抗纤维化作用，可改善肺功能并延缓AE-IPF的进展。然而，目前尚无相关临床试验数据，期待更多的临床研究进一步证实尼达尼布在延缓AE-IPF进展中的重要价值。

综上，ILD-LC患者应注重围术期的多学科管理，涵盖放射科、呼吸科等专科医师，参与ILD病情评估、围术期抗纤维化药物使用等。患者术前需进行全面的肺功能检查，包括肺容量测定和DLCO等，从而评估肺功能损害的严重程度，以判断患者是否具备手术指征。此外，在整个治疗过程中应定期复查肺功能，从而及时评估ILD的变化情况。同时，我们还建议定期监测静息和运动时的血氧水平，以评估是否需要吸氧治疗或调整氧流量。

## 5 小结与展望

本文系统梳理了ILD-LC的鉴别诊断、手术策略制定、围术期管理等内容，尤其对术后AE-ILD的风险识别及处理策略进行了详细阐述，以期为广大胸外科医师对ILD-LC的诊疗管理提供一定的借鉴。然而，鉴于ILD-LC本身的复杂性和患者个体差异，具体的手术策略制定及围术期管理仍需根据临床实际进行个体化决策。结合ILD-LC当前的研究成果，未来继续深入研究ILD-LC的发病机制以发现新的治疗靶点，运用多组学技术探索可预测AE-ILD发生风险及预后的标志物，开展多中心临床研究以期为ILD-LC手术策略的制定提供更多循证医学证据，以及开发新型抗纤维化药物应用于ILD-LC的围术期管理等，或可成为ILD-LC潜在的研究热点。
